# Lectures on Distortion of the Spine Not Connected with Caries, Delivered in the Theatre of St. George’s Hospital

**Published:** 1847-03

**Authors:** B. C. Brodie


*Lectures on Distortion of the Spine not connected with Caries, delivered in the Theatre of St. George's Hospital.* By Sir B. C. Brodie, Bart. F. R. S.—In a person of what is called a good figure, when the feet rest on the ground with the heels in contact, the lower limbs being straight, and the upper limbs occupying the same position on the sides, the spine rises perpendicularly in the same line with the *os sacrum*, and making on each side the same angle with the pelvis; the centre of gravity being somewhere in the middle line of the body. Whatever causes the centre of cavity to shift its place so as to be on one side of the central line, will necessarily cause some alteration in the position of the spine. A heavy weight in one pocket, or held in one hand, where there is none in the other; the greater height of the heel of one shoe, or an unequal leaning on the two lower limbs: even such trivial matters as these will occasion more or less of lateral deviation of the spine. If from any cause whatever, the centre of gravity be moved far away from the spine, the individual would necessarily fall to the ground if he were not to make an effort to prevent it. The result of that effort is, that, while the cause first in operation, whatever that may be, produces a bending to one side in one part of the spine, by the action of the muscles another part of the spine is bent in the opposite direction: and thus, in all cases in which a lateral curvature exists, the curvature is double, the whole spine assuming a form which is usually, and not very inaptly, compared to that of the italic *S*. The degree of such curvature of course varies in different cases. In many persons it is so trifling as to be scarcely perceptible, in others it is so considerable as to become a great and obvious deformity.

From whatever cause the spine becomes affected with a lateral curvature, it cannot but happen that the effects of it should extend to other parts of the trunk. The ribs corresponding to the convex side of the curvature are at their anterior extremities separated from each other by a wider interval than is natural, while on the opposite side they are, as it were, squeezed together, and compressed into a smaller compass. The *os ilium* of one side appear to be more prominent than that of the other; and there is a corresponding change in the appearance of the scapula, and clavicles, and even of the sternum. I notice these changes only briefly at present; I shall explain them more particularly hereafter.

No part of the body can be permanently displaced without undergoing an alteration in its figure. In a case of unreduced dislocation of the humerus, the old glenoid cavity is absorbed, and a new articulating surface is generated on the lower margin of the scapula, while the head of the humerus becomes reduced in size, and otherwise altered so as to correspond to the parts with which it is now in apposition. In like manner, in an established case of lateral curvature of the spine, the bodies of the vertebræ are found reduced in thickness on one side, increased in thickness on the other; the ribs bulge unnaturally in one place, and are unnaturally depressed in another: and corresponding changes takes place in the clavicles and sternum, and even in the scapulæ. These changes are produced the more readily, because when lateral curvature exists it almost invariably begins to show itself in early life, while the process of growth is going on. These facts should be borne in mind, with a reference to practice. As deviations from the natural figure occur with more facility during the period of growth, so is the restoration of the natural figure more easily obtained during the same period also. The treatment of the disease cannot be begun too soon after the first signs of spinal curvature are perceptible, and little or no benefit can arise from the continuance of the treatment after the period of growth is completed.

Having made these preliminary observations, I am better enabled to explain to you the various circumstances in which lateral curvature of the spine may have its origin.

If one lower limb be shorter than the other, the ilium on that side (both feet being planted equally on the ground) must necessarily be depressed, and a double curvature of the spine is the consequence, produced in the manner which I have already mentioned; that is, one by the altered position of the pelvis, the other by the muscular effort which the patient makes to keep the centre of gravity in its proper place.

A difference in the length of the two limbs is in some instances the result of original formation. I have had young persons brought to me because one shoulder was observed to be higher than the other,

(this being usually the first thing observed in the commencement of a lateral curvature of a spine,) and in whom I have found the *femur and tibia* on one side respectively shorter than the *femur and tibia* of the other, although there neither were at the time, nor had been previously, any indications of disease in either limb. At other times, however, the difference in the length of the femur or tibia is clearly to be traced to disease. A diseased bone may grow less rapidly than the corresponding bone which remains in a healthy state. Thus, in a case of scrofulous affection of the bones of one finger, it is very common for the finger thus affected not to grow at all, while the other fingers grow as usual. The reverse of this also may happen, and the diseased bone in certain cases becomes actually longer than its fellow of the opposite side. So it was in two cases of necrosis of the femur, of which I have preserved notes, and which had in consequence been mistaken for cases of disease of the hip-joint. Now, in such cases as these which I have just described, it must be evident to you that the only thing to be done is to endeavor to equalise the length of the limbs by making the sole of one shoe thicker than that of the other. Nothing done to the spine itself can be of the smallest service. Indeed, for the most part, in these cases, the curvature of the spine is trifling. The addition of a very little cork to the sole of one shoe will be sufficient to prevent its being observed at all: and a clever shoemaker will easily manage so that the difference of the two shoes will be imperceptible also.

Those last observations will apply equally to another class of cases, in which, after fracture of the femur, or even of the tibia, the limb is shortened, and curvature of the spine is the consequence. Nothing done to the limb can restore its proper length, and nothing done to the spine itself can restore its proper figure. A thick sole to the shoe is the only remedy.

In a young person who has recovered from disease of the hip-joint after the formation of abscess, (and in some cases even where suppuration has not taken place,) the limb on the side of the disease is left considerably shortened. The shortening of the limb is sometimes the consequence of actual dislocation; at other times it arises from the margin of the acetabulum having been destroyed by ulceration, or from a partial destruction of the head of the femur, the limb being afterwards drawn upward by the action of the glutæi muscles. In whatever way the shortening of the limb is produced, it necessarily happens that as soon as the patient begins to walk the spine becomes distorted. There is, however, in many of these cases, another cause operating so as to produce the same result. The patient has been lying for a long time in bed without any particular attention being paid to the position in which he has placed himself, and this position has probably been that of lying on one side with the spine twisted laterally. The lateral curvature thus produced of course continues to exist when the patient first begins to stand and walk. Now of these two kinds of curvature the first is evidently irremediable; but the latter admits of considerable, and perhaps of complete, relief, under a simple mode of treatment, which I shall explain to you hereafter.

There is a peculiar paralytic affection to which children are liable, and which I have in my lectures been accustomed to describe under the name of *infantile paralysis*. The child (generally after suffering from an attack of fever,) exhibits symptoms of what is commonly called "determination of blood," to the brain and not improbably has an attack of convulsions. Then, all at once the muscles of some part of the body lose their power of acting under the influence of the will. In a few cases, recourse being immediately had to the exhibition of mercury, the paralysis is relieved. In the majority of cases it is not relieved at all, but remains unaltered through the rest of the patient's life. Now, if this has happened in one of the lower limbs, you need only refer to the observations which I made in the beginning of the lecture to be satisfied that a lateral curvature of the spine must be the consequence. One lower limb will support the weight of the body, the other will not support it: one limb is heavier than the other, and one limb only is exercised. The pelvis under these circumstances must become depressed on one side more than on the other, and a lateral inclination of the spine will follow depression of the pelvis. In some instances all the muscles of the leg and thigh are paralytic, and the whole of the lower limb is useless to the patient. In other cases perhaps not more than one or two muscles are thus affected, and the curvature of the spine varies accordingly.

In a very few of such cases, where the paralysis is of limited extent, the application of an instrument which in some degree supplies the place of the muscles whose power is deficient may be useful in assisting the patient to retain the erect posture. In other cases, where, in consequence of the want of power in the antagonist muscles those of the calf of the leg are contracted, the heel being elevated so that it cannot be brought into contact with the ground, some good may be done by the subcutaneous division of the *tendo Achillis*. But the advantage obtained in either of these ways is of limited extent, and beyond what I have just mentioned nothing is to be expected from the exercise of our art.

The effect of paralysis on the figure of the spine is not confined to those cases in which the seat of the paralysis is in the lower limbs. Even a *partial* loss of power in the muscles of one of the upper limbs will, in a growing person, become a cause of spinal curvature, and in one case which fell under my observation, in which there had been, from infancy, a complete paralysis of *all* the muscles of the arm, forearm, and hand, the spine was as much distorted as it would have been in a case of recovery from diseased hip-joint with a very contracted limb. The distortion here is to be attributed to the difference in the weight of the two limbs, and the greater muscular action on the side opposite to that of the disease, combining to draw the centre of gravity out of the middle line of the body. Of course the case is beyond the reach of remedies. Nothing can restore the spine to its proper condition but the removal of the paralysis, a thing rarely to be accomplished, even when you are consulted in the first instance, and of which there certainly can be no reasonable expectation at that later period when the attention of the parent is first called to the alteration of the patient's figure.

Another cause of lateral curvature of the spine is a difference in the capacity of the two sides of the chest. Hypertrophy of the heart, or a diminution in the size of one lung, will produce the same effect. I was consulted concerning a little girl in whom there was an unusual degree of this kind of distortion. On examination I found that she breathed with one lung only, and that the other side of the chest was reduced to a very small size, the ribs lying almost in contact with each other. The fact proved to be, that, two or three years before the period of my being consulted, she had suffered from a severe attack of pneumonia, in consequence of which the organization of one lung had been completely destroyed, so as to render it altogether useless, respiration being performed wholly by the other. Some mechanical apparatus had been recommended in this case with a view to relieve the spinal curvature; but I need scarcely explain to you why neither this nor any other kind of treatment can, under such circumstances, be of the smallest service.

It was the prevailing opinion formerly, and I believe that some hold the opinion still, that the common cause of a lateral curvature of the spine is a rickety condition of the bones. This view of the pathology of the disease is, however, not confirmed by the specimens preserved in museums of morbid anatomy, and no one who has seen much of these cases in the living person can doubt that the fact is otherwise. The altered shape of rickety bones, in which there is, as you well know, a deficiency of hard earthy matter (phosphate of lime) depends partly on the action of the muscles, but still more on the operation of the superincumbent weight. The greater the weight the greater is the distortion. Hence in a rickety child the disease is manifested first in the legs, then in the thighs, then in the pelvis, and afterwards in the spine. Now, in cases of lateral curvature of the spine, it rarely happens that there is anything like the rickety flexure of the lower limbs. In the rickety pelvis the two *ossa pubis* are as it were squeezed towards each other both behind and below the symphysis. The form of the brim of the pelvis is altered, the diameter of it being diminished in the direction from before backwards, and increased from side to side. One result of such distortion of the pelvis is, that parturition, at the full period of utero-gestation, is rendered either difficult or impossible. Nevertheless we meet with instances without number of women with very considerable lateral curvature of the spine who have born children with as little inconvenience as others whose spines were straight.

We are not, therefore, justified in regarding rickets as the common, or even as a frequent cause of spinal curvature: nevertheless, it is the cause of it in a few instances. The curvature in these cases is, for the most part, not merely lateral, but there is a bending of the lower part of the spine forward, so that the spinous processes project posteriorly in the form of a segment of a circle. This circumstance, and the condition of the legs and thighs, afford sufficient grounds for our diagnosis.

What is to be said as to the treatment of this variety of distorted spine may be comprised in a few words, and will be more conveniently introduced here than in any other place. I know of no reason why the treatment of the rickety affection of the spine should be different from that of the rickety affection of the legs and thighs. Of this last I see a great number of cases. In a large proportion of them, heavy instruments of steel have been already applied, with a view to reduce the curvature. In others the same thing has been recommended either by instrument makers or surgeons, but the machinery has not yet been applied. Now what is the effect of this mode of treatment? The original curvature is probably removed, but in order that this object should be attained, the instrument must make pressure on at least two points, one in the limb above, and the other in the limb below, and at each of these points a curvature is produced which did not exist before, so that there is simply an exchange of one curvature for two others. Then the instruments are a great weight and encumbrance to the child. He cannot drag them about so as to take such an amount of exercise as is necessary for the maintenance of the general health. They harass and torment him; and as they are always liable to break, and be otherwise out of repair, they are an endless trouble and expense to the parents. There is only one form of the disease in which, according to my experience, the use of instruments is at all justifiable, and that is one of very rare occurrence, in which the flexure is confined to the superior epiphysis of the tibia, the tibia below the epiphysis being bent outwards, making an angle more or less obtuse with the femur, so that the sole of the foot is with difficulty placed on the ground. With this single exception, I have not seen a single case of rickety curvature of the lower limbs, in which, if the health could be improved, and the general vigour of the system maintained, the curvature did not disappear spontaneously without any kind of local treatment being had recourse to; while, on the other hand, under a continuance of bad health, every kind of local treatment has been ineffectual. I generally recommend that the child should live in the country rather than in a crowded city; that he should be as much as possible at the sea-side; that he should take some preparation of iron from time to time, the bowels being at the same time carefully regulated: that he should use a shower-bath every morning, cold in summer, with the chill taken off in winter; and that he should live on a plain but nutritious diet. In the early part of my practice I advised that he should be encouraged to crawl on the floor rather than to use his feet, and that instead of running about out of doors he should be taken into the fresh air in an open carriage. I am now convinced that this advice was wrong; that the general health cannot be maintained without exercise; that the more the limbs are used the better chance is there of the necessary quantity of phosphate of lime being deposited in the bones; and that as the bones become harder so will they most certainly regain their proper figure, in spite of the weight which they have to sustain. Even in what might be termed a bad case of rickety affection of the limbs, three or four years, and in slighter cases a still shorter period, will generally be sufficient for this beneficial change to be brought about. From what I have already said, you may be aware that I have a more limited experience of rickety disease as it exists in the spine than as it exists in the extremities; but nevertheless I have seen enough of it to be satisfied that the plan of treatment which is the best adapted in the one case is also the best adapted to the other.—*Lon. Med. Jour.*

